# P-1080. Aztreonam-Avibactam Activity Against Gram-negative Bacteria Isolated from Patients with Pneumonia from Europe, Asia, and Latin America (2021–2023)

**DOI:** 10.1093/ofid/ofae631.1268

**Published:** 2025-01-29

**Authors:** Helio S Sader, Hank Kimbrough, S J Ryan Arends, Marisa Winkler, Rodrigo E Mendes, Mariana Castanheira

**Affiliations:** JMI Laboratories, North Liberty, Iowa; Element Materials Technology/Jones Microbiology Institute, North Liberty, Iowa; JMI Laboratories / Element, North Liberty, Iowa; Element Materials Technology/Jones Microbiology Institute, North Liberty, Iowa; JMI Laboratories, North Liberty, Iowa; JMI Laboratories, North Liberty, Iowa

## Abstract

**Background:**

Aztreonam-avibactam (ATM-AVI) is under development to treat Gram-negative (GN) infections. We evaluated the antimicrobial susceptibility of GN bacilli causing pneumonia

Activity of β-lactamase inhibitor combinations

**Figure:**
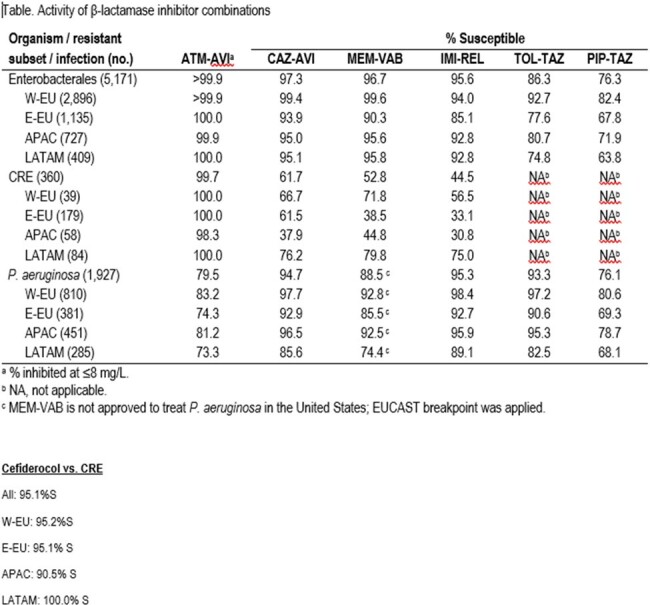

**Methods:**

7,560 organisms were consecutively collected (1/patient) from patients with pneumonia in 59 medical centres located in Western Europe (W-EU; 10 countries; 24 centres; 3,977 isolates), Eastern Europe (E-EU; 9 countries; 15 centres; 1,617 isolates), the Asia-Pacific region (APAC; 7 countries; 12 centres; 1,238 isolates), and Latin America (LATAM; 6 countries; 8 centres; 728 isolates). Isolates were susceptibility tested by broth microdilution. Cefiderocol was only tested against carbapenem-resistant Enterobacterales (CRE). CLSI breakpoints were applied. An ATM-AVI PK/PD susceptible (S) breakpoint of ≤8 mg/L was applied for comparison. CREs were screened for carbapenemase-encoding genes by whole genome sequencing.

**Results:**

ATM-AVI inhibited >99.9% of Enterobacterales (ENT) at ≤8 mg/L (MIC_50/90_, 0.06/0.25 mg/L) and showed consistent activity across regions (Table). Only 2 ENT had an ATM-AVI MIC >8 mg/L, an *E. cloacae* (Italy) and a *K. pneumoniae* (Taiwan). CRE rates ranged from 1.3% (W-EU) to 20.5% (LATAM). Multidrug-resistance (MDR; non-S to ≥3 classes) rates varied from 20.2% (W-EU) to 47.2% (LATAM). ATM-AVI retained potent activity against CRE (MIC_50/90_, 0.25/0.5 mg/L; 99.7% inhibited at ≤8 mg/L) and MDR ENT (MIC_50/90_, 0.12/0.5 mg/L; 99.9% inhibited at ≤8 mg/L). Cefiderocol was active against 95.8% of CRE. Ceftazidime-avibactam (CAZ-AVI; 61.7% S), meropenem-vaborbactam (MEM-VAB; 52.8%S), and imipenem-relebactam (IMI-REL; 44.5% S) showed limited activity against CRE and S rates varied greatly among regions. A metallo-β-lactamase was identified in 35.8% of CREs, varying from 23.8% in LATAM to 60.3% in APAC. IMI-REL (95.3% S), CAZ-AVI (94.7% S), and ceftolozane-tazobactam (TOL-TAZ; 93.3% S) were the most active agents against *P. aeruginosa*; whereas ATM-AVI inhibited 79.5% of isolates at ≤8 mg/L and 76.1% were piperacillin-tazobactam (PIP-TAZ)-S. ATM-AVI inhibited 98.9% of *S. maltophilia* isolates (n=440) at ≤8 mg/L.

**Conclusion:**

ATM-AVI was the most active compound against CRE and *S. maltophilia* isolated from patients with pneumonia.

**Disclosures:**

**Marisa Winkler, MD, PhD**, Element Iowa City (JMI Laboratories) was contracted to perform services in 2023 for > 30 biotech and pharmaceutical companies: Grant/Research Support **Rodrigo E. Mendes, PhD**, GSK: Grant/Research Support

